# Association between *Osteopontin* Promoter Gene Polymorphisms and Haplotypes with Risk of Diabetic Nephropathy

**DOI:** 10.3390/jcm4061281

**Published:** 2015-06-10

**Authors:** Balneek Singh Cheema, Sreenivasa Iyengar, Rajni Sharma, Harbir Singh Kohli, Anil Bhansali, Madhu Khullar

**Affiliations:** 1 Department of Experimental Medicine and Biotechnology, Postgraduate Institute of Medical Education and Research, Chandigarh 160012, India; E-Mails: balneekcheema@gmail.com (B.S.C.); rajni.sharma28@gmail.com (R.S.); 2 Department of Nephrology, Postgraduate Institute of Medical Education and Research, Chandigarh 160012, India; E-Mails: sreeni08@gmail.com (S.I.); kohlihs2009@gmail.com (H.S.K.); 3 Department of Endocrinology, Postgraduate Institute of Medical Education and Research, Chandigarh 160012, India; E-Mail: anilbhansali_endocrine@rediffmail.com

**Keywords:** diabetic nephropathy, haplotypes, *Osteopontin* promoter gene polymorphism, type 2 diabetes, eGFR

## Abstract

Background: *Osteopontin* (*OPN*) C-443T promoter polymorphism has been shown as a genetic risk factor for diabetic nephropathy (DN) in type 2 diabetic patients (T2D). Methods: In the present study we investigated the association of three functional promoter gene polymorphisms C-443T, delG-156G, and G-66T and their haplotypes with the risk of DN and estimated Glomerular Filtration Rate (eGFR) in Asian Indians T2D patients using Real time PCR based Taqman assay. A total of 1165 T2D patients, belonging to two independently ascertained Indian Asian cohorts, were genotyped for three *OPN* promoter polymorphisms C-443T (rs11730582), delG-156G (rs17524488) and G-66T (rs28357094). Results: -156G allele and GG genotypes (delG-156G) and haplotypes G-C-G and T-C-G (G-66T, C-443T, delG-156G) were associated with decreased risk of DN and higher eGFR. Haplotype G-T-delG and T-T-delG (G-66T, C-443T, delG-156G) were identified as risk haplotypes, as shown by lower eGFR. Conclusion: This is the first study to report an association of *OPN* promoter gene polymorphisms; G-66T and delG-156G and their haplotypes with DN in T2D. Our results suggest an association between *OPN* promoter gene polymorphisms and their haplotypes with DN.

## 1. Introduction

Diabetic nephropathy (DN) is the leading cause of end-stage renal disease worldwide and approximately 30% of type 2 diabetic patients (T2D) develop DN irrespective of glycemic control [1]. In addition to the effect of environmental factors, there is abundant evidence in support of genetic susceptibility to DN in individuals with both type 1 diabetes mellitus (T1DM) and T2DM [2,3].

Recently, osteopontin (*OPN*), a large phosphoglycoprotein adhesion molecule, has emerged as a potential pathophysiologic contributor in DN. Osteopontin has also been found to be associated with renal diseases characterized by macrophage infiltration, tubulointerstitial fibrosis, and proteinuria [4,5,6]. Genetic polymorphisms in promoter region of the *OPN* gene have been shown to affect its transcription and expression and thus may be associated with disease susceptibility [7]. Giacopelli *et* *al.* reported that three functional polymorphisms in *OPN* promoter region (G-66T, delG-156G, and C-443T) modulates *OPN* transcription and expression; delG-156G and G-66T polymorphism affects the binding of RUNX2 binding site and SP1/SP3 binding site, respectively, to the *OPN* promoter region, leading to altered transcriptional activity [7].

Several recent studies have reported association of *OPN* gene variants with various renal diseases [8,9,10]. We have also previously found a modest association between *OPN* C-443T promoter gene polymorphism and increased risk of DN in T2D [11]. Functional genomics studies on *OPN* gene polymorphisms suggest that specific *OPN* haplotypes affect its expression more profoundly as compared to individual genotypes; for example, a specific *OPN* haplotype (G-66T, delG-156G, and C-443T) was found to confer a significantly reduced level of reporter gene expression [7]. Further, Hummelshoj *et al.* [12] showed a sequence specific binding of transcription factor SP1 with -66T allele, but not with -66G allele, and haplotype -443C/-156delG/-66T showed a marked increase in promoter activity of a luciferase reporter gene. Thus, it has been suggested that *OPN* haplotypes instead of single nucleotide polymorphisms may be a better predictor of genetic susceptibility and will allow achieving more accurate results. A few recent studies have confirmed *OPN* haplotypes as modifiers of disease susceptibility in sarcoidosis, nephrolithiasis, pseudoxanthomaelasticum, gliomas, Crohn’s disease, and oral carcinogenesis [9, 13,14,15,16,17]. In the present study we have investigated the association of three functional promoter gene polymorphisms C-443T, delG-156G, and G-66T and their haplotypes with the risk of DN in two independent cohorts of north Indian T2D patients. We also checked the association between *OPN* Single Nucleotide Polymorphisms (SNPs) and risk of DN and estimated Glomerular Filtration Rate (eGFR).

## 2. Subjects and Methods

### 2.1. Study Population

Two independently ascertained T2D cohorts of north Indian origin, visiting Endocrinology and Nephrology clinics of the Postgraduate Institute of Medical Education and Research, Chandigarh, between June 2006 to September 2007 (Cohort1) and January 2010 to March 2012 (Cohort 2) were recruited in this study. Cohort 1 consisted of 240 patients with DN and 255 patients with T2D; cohort 2 consisted of 455 patients with DN and 215 patients with T2D. Their ethnicity was confirmed on the basis of language spoken and ancestral history. The study was approved by Post Graduate Institute of Medical Education and Research, Chandigarh, ethics committee and written consent was obtained from participating subjects (MS/1304/DM/810). All the subjects were age, sex, and ethnicity matched, and had same mean duration of T2D (duration of onset of 5 years or more). We had ethnically homogeneous diabetic subjects who were enrolled from a single center, thus avoiding phenotyping errors and bias. They were two independent well-ascertained cohorts from a single Centre and were part of a homogeneous ethnic population. Time since diagnosis of T2D (years) in DM patients; Cohort 1: 15.6 ± 5.24, Cohort 2: 15.1 ± 6.3, HbA1c (%) in DM patients; Cohort 1: 7.6 ± 1.1, Cohort 2: 7.8 ± 1.4. Age; Cohort 1: DM: 58.10 ± 8.1, DN: 60.1 ± 6.1, Cohort 2: DM: 61.9 ± 8.6, DN: 54.1 ± 8.1, Gender; Cohort 1: DM: 105/150, DN: 94/146, Cohort 2: DM: 99/116, DN: 271/184, BMI; Cohort 1: DM: 23.9 ± 2.8, DN: 27.8 ± 2.9, Cohort 2: DM: 21.7 ± 4.4, DN: 24.1 ± 4.2.

T2D patients were divided into two groups according to the following diagnostic criteria: (1) cases of DN, that is, patients having age at onset of diabetes >35 years with T2D (duration of onset of 5 years or more) and DN, DN was defined as (a) 24 hproteinexcretion >500 mg and or, (b) anurinealbumin: creatinine ratio >300 µg/mg without any clinical or laboratory evidence of other kidney disease. (2) control, patients having age at onset of diabetes >35 years with T2D (duration of onset of 5 years or more), but showing normal urinary albumin excretion, that is, AER <20 μg/min. Urine sample was measured on two consecutive occasions. Urine dipstick analysis to determine pathological changes in a patient’s urine in standard urinalysis was performed for the analysis of blood, ketones, glucose, pH, bilirubin, urobilinogen and protein in urine sample.

Diabetic retinopathy (DR) was diagnosed by dilating the pupils with mydriatic, and then carefully examining the retina. Retinal photography or fluorescein angiography tests were also performed. About 77% of type 2 diabetics with nephropathy had retinopathy as compared to 34% of diabetics without nephropathy.

Notably, we excluded patients with end stage renal disease end stage renal disease (ESRD) from our study.

Patients with T1D, having any known non-diabetic renal disease and nephropathy other than DN were excluded from the study. Additionally, DN subjects who had microscopic hematuria, taking antihypertensive drug treatment and microalbuminuric patients were excluded from the study group to avoid misclassification of phenotype. The evidence of nephropathy was firmly reliable based upon confounding factors and continuous real-time assessment.

### 2.2. Genotyping

Genomic DNA was isolated from peripheral blood lymphocytes using proteinase K digestion and phenol chloroform method. The *OPN* promoter polymorphism C-443T (rs11730582), delG-156G (rs17524488) (Position: 13444429, NCBI Reference Sequence: NT_016354.19) and G-66T (rs28357094) (Position: 13444518, NCBI Reference Sequence: NT_016354.19) was determined using Real time PCR based Taqman assay (Applied Biosystems, Foster City, CA, USA) following manufacturer’s instructions. Positive and negative controls were used in each genotyping run, and 5% of randomly selected samples were re-genotyped by other lab personnel with 100% concordance. The genotypes were also confirmed by randomly sequencing some of the samples. Symbol: *SPP1*, Full Name: secreted phosphoprotein 1, also known as *OPN*.

### 2.3. Statistical Analysis

The statistical tests were performed, using the SPSS Inc., Chicago, IL version 11.0. We tested the genotype and allele frequencies for deviation from Hardy-Weinberg equilibrium (HWE) proportions by using an HWE calculator [18]. Using a chi-squared test the genotype distribution for HWE was considered significant at *p* < 0.05. Significant allelic and genotypic associations calculated by Pearson’s χ^2^-test were used for evaluating odds ratio (OR) and 95% confidence intervals (CI). Bonferroni’s correction was applied to the p-values that were considered significant when *p* < 0.05. Multivariate logistic regression was used to compute odds ratio for developing DN by adjusting for potential confounders which include age, sex, BMI, systolic blood pressure, smoking, duration of diabetes, duration of hypertension and HbA1c.Power analysis was performed using Quanto (version 1.2; http://hydra.usc.edu/gxe) and was calculated by defining the region of acceptance and effect size. Haplotype frequencies were estimated in various subject groups with the help of Phase Ver 2.1 software [19]. Finally, statistical analysis was performed to determine significance and risk ratio between haplotyped groups. Linkage disequilibria (LD) were also estimated in the study population using Haploview software [20].

## 3. Results

### 3.1. Association between OPN SNPs and Risk of DN and Estimated GFR

Genotype and allele frequencies of the *OPN* delG-156G, G-66T and C-443T polymorphism in both cohorts (1, 2) are given in Table 1. The genotype frequencies were in HWE for both the cohorts for delG-156G, and G-66T polymorphism (*p* > 0.05). Significant deviation from HWE of genotype distribution was observed in the present population in C-443T in both DM and DN patients. Significant deviation from HWE was also observed in 200 healthy subjects without diabetes or other co morbidities in C-443T (CC:CT:TT = 166:28:6; *p* = 0.002). We observed decreased risk of DN among carriers of delG-156G (GG) genotype in both cohorts (Table 1); patients with the GG genotype also showed highest estimated GFR (Table 2). G-66T polymorphism showed no significant association with DN and estimated GFR in any of the two cohorts (Table 1 and Table 2).

**Table 1:** Allele and genotype frequency of *OPN* gene promoter polymorphism in subjects with type 2 diabetes mellitus (T2D) *vs.* diabetic nephropathy (DN).

delG-156G	Cohort 1			Cohort 2			
	T2DM (*n =* 255)	DN (*n =* 240)	Adjusted OR (95% CI) *p* *	T2DM (*n =* 215)	DN (*n =* 455)		Adjusted OR (95% CI) *p* *
**Allele Frequency**	delG = 288 (0.56)	delG = 347 (0.72)	**0.69 (0.58–0.85) <0.00001**	delG = 237 (0.55)	delG = 656 (0.72)		**0.68 (0.58–0.81) <0.0001**
	G = 222 (0.44)	G = 133 (0.28)		G = 193 (0.45)	G = 254 (0.28)		
**Genotype Frequency**	delGdelG = 84 (0.33) delG G = 120 (0.47) GG = 51 (0.20) delG G + GG = 171 (0.67)	delGdelG = 122 (0.51) delG G = 103 (0.43) GG = 15 (0.06) delG G + GG = 118 (0.49)	**0.79 (0.50–0.97) 0.009** **0.40 (0.21–0.58) <0.0001** **0.68 (0.53–0.88) <0.0001**	delGdelG = 67 (0.31) delG G = 103 (0.48) GG = 45 (0.21) delG G + GG = 148 (0.69)	delGdelG = 228 (0.50) delG G = 200 (0.44) GG = 27 (0.06) delG G + GG = 227 (0.50)		**0.77 (0.60–0.97) 0.003** **0.38 (0.31–0.52) <0.0001** **0.65 (0.52–0.83) <0.0001**
**G-66T**							
**Allele Frequency**	G = 383 (0.75)	G = 350 (0.73)	0.97 (0.72–1.30) 0.88	G = 313 (0.73)	G = 683 (0.75)	-	
	T = 127 (0.25)	T = 130 (0.27)		T = 117 (0.27)	T = 227 (0.25)	0.88 (0.68–1.14) 0.37	
**Genotype Frequency**	GG = 138 (0.54) GT = 107 (0.42) TT = 10 (0.04) GT + TT = 117 (0.46)	GG = 127 (0.53) GT = 96 (0.40) TT = 17 (0.07) GT + TT = 113 (0.47)	1.07 (0.75–1.54) 0.58 0.98 (0.71–1.63) 0.31 1.03 (0.72–1.47) 0.92	GG = 109 (0.51) GT = 95 (0.44) TT = 11 (0.05) GT + TT = 106 (0.49)	GG = 255 (0.56) GT = 173 (0.38) TT = 27 (0.06) GT + TT = 200 (0.44)	0.79 (0.56–1.10) 0.17 0.97 (0.56–1.48) 1 0.81 (0.58–1.12) 0.23	
**C-443T**							
**Allele Frequency**	C = 454 (0.89)	C = 360 (0.75)	**2.68 (1.87–3.84) <0.0001**	C = 378 (0.88)	C = 673 (0.74)	**1.59 (1.12–2.50) <0.0001**	
	T = 56 (0.11)	T = 120 (0.25)		T = 52 (0.12)	T = 237 (0.26)		
**Genotype Frequency**	CC = 206 (0.81) CT = 41 (0.16)	CC = 151 (0.63) CT = 58 (0.24)	**1.96 (1.22–3.13) 0.007**	CC = 172 (0.80) CT = 32 (0.15)	CC = 278 (0.61) CT = 118 (0.26)	**1.69 (1.18–3.11) 0.0002**	
	TT = 8 (0.03)	TT = 31 (0.13)	**3.78 (2.05–3.13) 0.0001**	TT = 11 ( 0.05)	TT = 59 (0.13)	**2.04 (1.59–2.89) 0.0003**	
	CT + TT = 49 (0.19)	CT + TT = 89 (0.37)	**1.98 (1.65–2.72) 0.0001**	CT + TT = 43 (0.20)	CT + TT = 177 (0.39)	**1.54 (1.28–2.50) 0.0002**	

The *p* values for the models are adjusted for confounding factors including age, sex, BMI, systolic blood pressure, smoking, duration of diabetes, duration of hypertension and HbA1c * *p* < 0.05.

**Table 2:** The estimated Glomerular Filtration Rate (eGFR) values of study subjects according to *Osteopontin* (*OPN*) (delG-156G) genotype.

	Cohort 1				Cohort 2			
	delGdelG (122)	delG G (103)	GG (15)	*p*	delGdelG (228)	delG G (200)	GG (47)	*p*
**eGFR**	46.1 ± 23.4	51.4 ± 20.2	66.8 ± 22.0	0.008	44.8 ± 22.9	49 ± 21.1	67.2 ± 21.6	0.009

Data are mean ± SD; *p*-values of *p* < 0.05 were adjusted for age, sex, BMI and duration of diabetes.

### 3.2. Haplotype Analysis

Seven major *OPN* haplotypes (with frequency >5%) were observed in both cohorts. The frequency of haplotypes G-T-delG and T-T-delG (allele of G-66T, T-443C, and delG-156G) was significantly higher in DN group than in T2D and was associated with nearly 1.5-fold increased risk of DN (Table 3) and lower eGFR (Table 4) in both cohorts. The frequency of haplotypes G-C-G and T-C-G (allele of G-66T, C-443T, and delG-156G) was significantly lower in patients with DN, and were associated with a nearly 60% decreased risk of DN (Table 3) and a higher eGFR (Table 4) in both cohorts as compared to G-C-delG haplotype (allele of G-66T, C-443T, and delG-156G).

### 3.3. Linkage Disequilibria (LD) Analysis

LD values were generated to look for association among the three studied polymorphisms. No significant LD was observed among the polymorphisms (G-66T, C-443T, and delG-156G; *p* > 0.05). (Table 5).

### 3.4. Comparison between Genotypes and Haplotypes

The G allele of *OPN* delG-156G promoter polymorphism, individually, was associated with approximately 40% decreased risk of DN, whereas G-C-G and T-C-G haplotype (G allele of delG-156G) was associated with 60% decreased risk of DN, in both the cohorts. T allele of C-443T allele individually was associated with approximately 2.5-fold increased risk of DN, whereas G-T-delG and T-T-delG haplotype (T allele of C-443T) was associated with 1.5-fold increased risk of DN, in both cohorts.

**Table 3:** *Osteopontin* (*OPN*) promoter gene haplotype frequency distribution in type 2 diabetes mellitus (T2D) *vs.* diabetic nephropathy (DN).

	Cohort 1				Cohort 2			
**Haplotype**	DN (2*n* = 480)	T2DM (2*n* = 510)	p *	OR 95% CI	DN (2*n* = 910)	T2DM (2*n* = 430)	*p* *	OR 95% CI
**G-C-delG**	62 (0.13)	62 (0.12)	Reference		100 (0.11)	44 (0.10)	Reference	
**G-T-G**	130 (0.27)	142 (0.28)	0.72	0.83 (0.45–1.39)	254 (0.28)	124 (0.29)	0.75	0.81 (0.51–1.12)
**G-T-delG**	**62 (0.13)**	**36 (0.07)**	**0.02**	**1.74 (1.12–2.81)**	**128 (0.14)**	**26 (0.06)**	**0.01**	**1.81 (1.19–2.87)**
**G-C-G**	**52 (0.11)**	**86 (0.17)**	**0.04**	**0.39 (0.20–0.74)**	**82 (0.09)**	**74 (0.17)**	**0.02**	**0.41 (0.23–0.68)**
**T-T-G**	20 (0.04)	16 (0.03)	0.95	1.04 (0.68–1.80)	28 (0.03)	16 (0.04)	0.81	0.97 (0.71–1.54)
**T-T-delG**	**62 (0.13)**	**40 (0.08)**	**0.02**	**1.70 (1.10–2.51)**	**128 (0.14)**	**38 (0.09)**	**0.02**	**1.58 (1.07–2.18)**
**T-C-G**	**30 (0.06)**	**52 (0.10)**	**0.007**	**0.38 (0.23–0.70)**	**54 (0.06)**	**52 (0.12)**	**0.008**	**0.41 (0.24–0.64)**
**T-C-delG**	62 (0.13)	76 (0.15)	0.79	0.81 (0.56–1.3)	136 (0.15)	56 (0.13)	0.82	1.12 (0.71–1.28)

Order of SNPs in *OPN* gene haplotypes: (G-66T, C-443T, delG-156G); * *p* < 0.05 has been subjected to Bonferroni correction.

**Table 4:** The estimated Glomerular Filtration Rate (eGFR) values of study subjects according to *Osteopontin* (*OPN*) promoter gene haplotype.

	Cohort 1								
	G-C-delG (62)	G-T-delG (62)	T-T-delG (62)	G-C-G (52)	T-C-G (30)	G-C-delG	G-C-delG	G-C-delG	G-C-delG
						*vs*.	*vs*.	*vs*.	*vs*.
						G-T-delG	T-T-delG	G-C-G	T-C-G
						*p*	*p*	*p*	*p*
**eGFR**	46.1 ± 23.4	35.6 ± 20.0	35.8 ± 21.3	65.3 ± 21.6	65.9 ± 21.6	0.006	0.009	0.008	0.006
	**Cohort 2**								
	G-C-delG (100)	G-T-delG (128)	T-T-delG (128)	G-C-G (82)	T-C-G (54)	G-C-delG	G-C-delG	G-C-delG	G-C-delG
						*vs*.	*vs*.	*vs*.	*vs*.
						G-T-delG	T-T-delG	G-C-G	T-C-G
						*p*	*p*	*p*	*p*
**eGFR**	45.8 ± 22.9	36.0 ± 19.8	35.7 ± 20.7	66.0 ± 20.7	65.3 ± 20.8	0.01	0.009	0.01	0.009

Data are mean ± SD; *p* values of *p* < 0.05 were adjusted for age, sex, BMI and duration of diabetes.

**Table 5:** A pair-wise comparisons of the polymorphisms, depicting the linkage disequilibria (LD) measures.

Variant 1	Variant 2	D′	LOD	r^2^
G-66T	C-443T	0.033	0.1	0.0010
G-66T	delG-156G	0.142	0.89	0.01
C-443T	delG-156G	0.01	0.0	0.0

D′, the coefficient of linkage disequilibrium; LOD, log of the odds of there being LD between two loci; r^2^, Correlation between a pair of loci.

## 4. Discussion

Functional genomics studies have identified three promoter region polymorphisms in *OPN* gene to affect its transcriptional activity and expression. These gene variants and their associated haplotypes have been found to predict better and more accurate disease association in several diseases [7]. We have previously shown that a promoter polymorphism in *OPN* (C-443T) increases the risk of DN in T2D patients [11]. In the present study, we investigated the association of two other *OPN* gene promoter polymorphisms (G-66T, delG-156G) and haplotypes of all three promoter polymorphisms with susceptibility to DN in Asian Indians. Our results showed that the G allele of *OPN* delG-156G promoter polymorphism was associated with decreased risk of DN and higher eGFR in two independently ascertained T2DM cohorts. Further, we identified two reduced risk-associated haplotype (G-C-G and T-C-G), which was associated with a nearly 60% decreased risk of DN and higher eGFR, and two increased risk-associated haplotype (G-T-delG and T-T-delG), which were associated with nearly 1.5-fold increased risk of DN and decreased eGFR. This is the first study to report association of *OPN* promoter gene polymorphisms, G-66T and delG-156G and their haplotypes, with DN in T2D. Modest association between these SNPs in the *OPN* promoter gene polymorphism with proteinuria and eGFR suggest that promoter SNPs in *OPN* might not only influence albuminuria but that this may also translate into a progressive deterioration of kidney function.

Significant deviation from HWE of genotype distribution in the present population in C-443T in both the cohorts may be due to moderate population size or the allele is rare which can cause a random change in allele frequencies. High frequency of mutation occurring at the specific loci can also cause deviation from HWE of genotype distribution in the present population. Moreover, we excluded the possibility of a typing error (LOD score > zero). Additionally, we screened one control group composed by 200 healthy subjects without diabetes or other co-morbidities to help us elucidate the plausible fact that Asian Indians are not in HWE for this variant.

A conditional analysis was performed to ensure that these are independent effects. Additionally, positive associations observed between *OPN* promoter gene polymorphism and their haplotypes with DN do not seem to be due to chance, as this association was replicated in two independent cohorts in our study and it persisted even after the influence of confounding factors was corrected. Moreover, the observed differences might be due to some population differences as North Indian population is an ethnically distinct population. Since we were aware that patients with longer duration of diabetes are at risk for long term problems including retinal, renal, cardiac and neurological problems, we reduced the duration of diabetes to five years, however, since all the patients were being exclusively followed up on at the institute for a long time, we turned up into same number of patients as were present in our earlier study [11] where the duration of disease for inclusion was 10 years.

We observed a consistently lower prevalence of G allele and GG genotype (*OPN*-156G) in the DN group as compared to the T2D group. *OPN*-156G allele has been earlier found to have a significant association with lower diastolic function in patients with diabetes mellitus, lower risk of developing calcium urolithiasis, more rapid progression of Duchenne muscular dystrophy, susceptibility to oral squamous cell carcinoma, systemic lupus erythematosus, glioma, and T1D [15,17, 21,22,23,24,25]. The increased *OPN* activity has been suggested to stimulate TGF-β and matrix deposition in mesangial cells, which could significantly contribute to pathophysiology of DN. Thus, decreased risk of DN among -156G allele and -156GG genotype observed in our study may be due to decreased *OPN* levels in these subjects. However, we could not measure *OPN* expression in these subjects as we did not have access to renal tissue from these subjects as renal biopsy from these subjects was not ethically approved. Recent evidence suggests that position -156 falls in a putative binding site for a component of the RUNX family of transcription factors [7]. Interestingly, SNPs in the RUNX binding site have been shown to be associated with macrophage infiltration, suggesting an important role of the -156G allele in DN.

Haplotypes G-C-G and T-C-G (60%) (G-66T, C-443T, delG-156G) were found to be a protective factor for DN, as shown by higher eGFR and Haplotype G-T-delG and T-T-delG (G-66T, C-443T, delG-156G) were identified as risk haplotypes as shown by a lower eGFR. This dual association of *OPN* haplotypes may be due to the differential effect of these haplotypes on *OPN* gene expression by influencing its promoter activity, as suggested by Giacopelli *et al*. [7], thus the genetic data align with the functional genomics data. Further, it has been proposed that variability in nucleotide sequences may also influence response to various stimuli and different factors involved in disease etiology. The exact molecular mechanisms by which *OPN* exerts its effects in DN are not well elucidated. Direct actions of *OPN* on mesangial cells represent important mechanisms by which *OPN* contributes to DN. Moreover, *OPN* participates in several processes, such as inflammation, regulation of inducible nitric oxide synthase in macrophages and renal tubular epithelial cells, renal epithelial cells apoptosis, modulation of macrophage adhesion, migration and cytokine release *etc.*, which are known to be involved in DN [26]. 

Our study has several strengths; our study had minimum power of 84% (power ranged from 80%–91% for selected polymorphisms of *OPN*) at a small effect size (0.1) and alpha level (0.05) and we confirmed our results in a replicative study in a similar homogeneous ethnic population. We selected subjects with the same mean duration of diabetes; moreover, multivariate logistic regression was used to compute odds ratio for developing DN by adjusting for potential confounders, which include age, sex, BMI, systolic blood pressure, smoking, duration of diabetes, duration of hypertension, and HbA1c. Although, we were aware that combining the cohorts would increase the statistical power, however, we were unable to merge the cohorts as they were independently ascertained T2D cohorts enrolled at different time periods. We chose these three SNPs for our study as we want to evaluate the effect of functional polymorphisms in *OPN* promoter region with risk of developing DN. Additionally, we were aware that SNPs G66T (rs28357094) and C443T (rs11730582) were in higher LD than in Asian Indian population (r^2^ = 0.373 and D′ = 1.0), which might be due to some population differences as the North Indian population is an ethnically distinct population. Our study fulfills most of the prerequisites for a good genetic association study as suggested by Bird *et al.* [27]. The limitation of our study was our inability to measure *OPN* activity in kidney tissues, as renal biopsies from these subjects was not ethically approved. Additionally, number of subjects might be too small to derive reliable conclusions from haplotype analysis, especially with minor haplotypes. A possible explanation for the fact as to why this locus, in spite having such a large effect, has not been seen in Genome Wide Association Studies (GWAS) may be due to the fact that no GWAS study has been done in an Asian Indian population, which is a genetically distinct population, exhibiting highest incidence of T2D, to date. The relatively medium size of our case-control study is a limitation that could introduce type 1 errors; however, very few investigators (mostly the ones involving a multicenter study) have access to a large sample size. However, sample size was predetermined for these variants to have a minimum power of 75%, which has been shown to be adequate for association studies. A small effect size (0.1) depicts ethnically homogeneous diabetic subjects, thus avoiding phenotyping errors and bias.

In summary, our results suggest association between *OPN* gene promoter polymorphisms and their haplotypes with risk of DN.

## 5. Conclusions

In this manuscript we are presenting data which show an association of *OPN* promoter gene polymorphisms; G-66T and delG-156G and their haplotypes with DN in T2D. Our results suggest an association between *OPN* promoter gene polymorphisms and their haplotypes with DN.
